# The Ongoing Evolution of Abdominal Aortic Surgery

**DOI:** 10.3390/jcm12010302

**Published:** 2022-12-30

**Authors:** Kyriakos Oikonomou, Carla Isabell Zimmler

**Affiliations:** Department of Vascular and Endovascular Surgery, Cardiovascular Surgery Clinic, University Hospital Frankfurt and Johann Wolfgang Goethe University Frankfurt, 60596 Frankfurt, Germany

Abdominal aortic surgery has witnessed significant paradigm shifts in recent years with the introduction of structured screening programs, as well as the evolution of endovascular aortic aneurysm repair (EVAR), which has allowed physicians to promptly identify and successfully treat an increasing number of patients, even including those previously considered unfit for open repair. The development of complex endovascular techniques has expanded our available treatment options in patients with hostile anatomic characteristics.

Nevertheless, many open questions remain regarding the pathophysiology and natural progression of the disease, as well as the long-term results of endovascular repair in comparison to open surgery, which is still considered the gold standard for repair by many. Especially following the recent pandemic and the limitations in hospital resources that it has generated, the focus turns to successfully predicting which patients have an increased risk for complications prior to and following aneurysm repair, and to tailoring the treatment modality, whether open or endovascular, to specific patient characteristics and needs.

To this end, the current Special Issue, with 17 accepted submissions in total, provides valuable insights into the field of pathogenesis and treatment of abdominal aortic aneurysm (AAA), highlighting outcome predictors and treatment options in the case of late repair failure.

New insights regarding the **pathogenesis of AAA** were proposed by the study of Behrens et al., which applied gene expression profiling in biopsies collected during open repair to highlight the heterogeneous gene expression patterns within the AAA wall, including genes involved in inflammation and ECM degradation [1].

Hauzer et al. examined the correlation of levels of calprotectin (CAL) and soluble receptors for advanced glycation end products (RAGE) which are thought to increase cytokine levels and inhibit matrix metalloproteinases with an AAA diameter. CAL levels appear to be a significant marker in patients with AAA. There is an almost twofold decrease in CAL levels after AAA excision [2].

Regarding AAA epidemiology, in an analysis of 3107 patients, Körfer et al. demonstrated that men and women present differences regarding aneurysm localization. Men had considerably more aneurysms in the abdominal aorta, common iliac artery, internal iliac artery, and popliteal artery, while women had a higher proportion of aneurysms in the ascending aorta, descending aorta, splenic artery, and renal artery. Following the detection of AAAs, clinicians are therefore encouraged to consider iliac and femoropopliteal extended ultrasound screening in men and thoracic cross-sectional imaging complementation in women [3].

In terms of clinically measurable progression parameters, blood pressure and pulse wave velocity (PWV), among others, should be considered. Schierling et al. evaluated the association between PWV and treated or untreated AoD, as well as other factors that may affect PWV. Aortic disease versus non-aortic AVD, its treatment, and cardiovascular risk factors (other than hypertension) had no significant effect on PWV. Successful blood pressure control is crucial to avoid high PWV and thus any increases in cardiovascular events [4].

This Special Issue highlights the **need for AAA treatment tailored to patient anatomy and pathology**. Ibrahim et al. investigated the value of open surgical repair (OSR) in the endovascular era and demonstrated that OSR is safe, effective, and durable in terms of the graft integrity and preservation of the renal function, even in patients with increased cardiovascular risk. COPD is a risk factor for mortality, leading to the conclusion that younger patients with long life expectancy and low perioperative risk may benefit from open repair [5]. Supporting this notion, the study of Barrena-Blázquez et al. showed that patients treated for AAA by EVAR and OSR had similar results on the quality-of-life SF-36 questionnaire in the short term. However, patients treated by EVAR had lower scores on the physical function, vitality, and mental health scales in the long term [6]. This factor should be taken into consideration alongside to the surgical success when planning a therapy for AAA.

When using EVAR, the use of arteriotomy closure devices (ACDs) has facilitated complete percutaneous access even when utilizing large-diameter sheaths. In a series of 376 puntures, Oikonomou et al. demonstrated that the application of a suture-mediated closure device is a safe and efficient method to achieve hemostasis, even in complex aortic pathologies, which require a staged approach with re-puncture [7].

A feared complication in aortic surgery, especially when extending the level of aortic repair above the celiac artery, is the occurrence of spinal cord ishaemia (SCI). The inhibition of proinflammatory cytokines by toll-like receptor 3 (TLR3) agonists and shock wave therapy has been investigated by Lobenwein et al. as a possible new therapeutic approach. Pre- and post-ischemic direct TLR3 activation as well as post-ischemic SWT can significantly decrease apoptosis and proinflammatory cytokine expression in vitro and might therefore pose possible new treatment strategies for ischemic spinal cord injury [8].

Late complications of OSR and EVAR include para-anastomotic aneurysm formation and aorto-enteric fistulas. Following the open surgical reconstruction of the infrarenal aorta, aneurysmal degeneration may occur at the level of the anastomosis. Fenestrated endovascular aneurysm repair (FEVAR) has been used to treat such aneurysms. The study by Marques de Marino et al. demonstrates a high technical success rate, low mortality and mobility rates, and a good mid-term outcome [9].

Secondary aorto-enteric fistulas following EVAR or OSR are complex pathologies requiring a very individualized therapeutic approach. In the study by Oikonomou et al. featuring 23 patients, all therapeutic approaches for this challenging pathology were associated with a high perioperative mortality rate. Patients who survived the perioperative period following open surgical repair showed acceptable midterm results during follow-up. In patients presenting with life-threatening bleeding, stent-graft implantation for bleeding control was associated with lower perioperative mortality rates [10].

Emergency EVAR for rupture is another specific clinical scenario associated with additional technical challenges. Van der Riet et al. retrospectively examined 74 patients treated with EVAR for a ruptured AAA (rAAA) and concluded that the post-operative diameter was significantly (*p* < 0.001) larger than the preoperative diameter at all aortic levels. The increase in the postoperative neck diameter correlates with preoperative hypotension. This discrepancy in neck aortic diameters can potentially lead to insufficient oversizing and insufficient lengths of endograft apposition, increasing the risk for a type IA endoleak; thus, it should be considered when oversizing the endograft in the case of rAAA [11].

Considering that AAA is a progressive disease and that late repair failure can be very challenging to treat, a focus of this Special Issue is determining and highlighting **predictors of late outcome**.

Ko et al. examined whether markers of inflammatory response and thrombus formation activation, such as the preoperative neutrophil-to-lymphocyte ratio (NLR), the platelet-to-lymphocyte ratio (PLR), or the mean platelet volume (MPV), can be used to predict 1-year mortality in patients undergoing open abdominal aortic aneurysm (AAA) repair. The authors demonstrated an independent relationship between the preoperative NLR and 1-year mortality in patients undergoing open AAA repair. Furthermore, the NLR and PLR had predictive power for 1-year mortality in ruptured cases [12].

Late outcome after EVAR is mostly influenced by preoperative anatomic characteristics. Veldhuizen et al. developed an innovative statistical shape model which provides a comprehensive quantitative description of the neck shape and may lead to new insights into the relationship between aortic neck morphology and EVAR outcomes in individual patients. Nine principal components provide a generalizable and robust model to determine the morphology of the infrarenal aortic neck [13]. By using 3D geometric analysis of balloon-expandable covered stents, authors from the same study group managed to better identify and more accurately classify possible complications following FEVAR [14].

The ultimate indicator for long-term EVAR success is aneurysm shrinkage. To this end, this Special Issue features two studies examining sack regression during follow-up (FU). Van Rijswijk et al. demonstrated that patients with a shrinking AAA, one year after EVAR, have better long-term survival compared to patients with a stable AAA size. Preoperative age, maximum AAA diameter, and infrarenal β angle showed to be useful in preoperatively differentiating patients with a shrinking AAA at a one-year follow-up from those with a stable AAA diameter. The final prediction model could correctly predict AAA shrinkage in two-thirds of the total patients [15]. In a study by Vedani et al., with a smaller patient population, no significant association between aneurysm shrinkage ≥5mm and survival or reintervention rates could be proven within one year [16]. These discrepancies underline the ongoing need for extensive prospective studies, utilizing additional imaging models and artificial intelligence.

Gray et al. further examined the changes in iliac anatomy following EVAR and their impact on the distal landing zone. There is a significant correlation between the oversizing and dilatation of the common iliac artery over time at the landing zone. Younger patients showed more rapid changes in the iliac vessel diameter in the first two years after EVAR. Almost 20% of the study collective showed detachment of the distal limb over time. Patients with a greater oversizing of the iliac limb showed lower limb detachment rates. Oversizing appears to be a key element in changes in the iliac anatomy, thus striking the right balance between sufficient oversizing to prevent detachment and not excessive oversizing that induces iliac dilatation must be found [17].

Despite the recent advances and paradigm shifts in treatment, AAA remains a challenging pathology with a variety of unknown underlying pathogenetic factors and an ongoing progression. Its treatment is associated with several technical challenges as well as dilemmas. The evolution of endovascular surgery has introduced an invaluable tool to our treatment armamentarium but cannot be considered panacea. The future of aortic surgery will be defined by our ability to identify the factors that are decisive for long-term success and thus enable us to tailor treatment modalities to individual patient characteristics.
